# Percutaneous bone marrow concentrate and platelet products versus exercise therapy for the treatment of rotator cuff tears: a randomized controlled, crossover trial with 2-year follow-up

**DOI:** 10.1186/s12891-024-07519-6

**Published:** 2024-05-18

**Authors:** Christopher J. Centeno, Zachary Fausel, Ehren Dodson, Dustin R. Berger, Neven J. Steinmetz

**Affiliations:** 1 Centeno-Schultz Clinic, Broomfield, CO 80021 USA; 2 Regenexx, LLC, Research and Development, Broomfield, CO 80021 USA

**Keywords:** Rotator cuff tears, Cell therapy, Bone marrow concentrate (BMC), Exercise therapy, Platelet-rich plasma (PRP), Musculoskeletal pain, Autologous orthobiologics, Shoulder

## Abstract

**Background:**

Surgical repair is recommended for the treatment of high-grade partial and full thickness rotator cuff tears, although evidence shows surgery is not necessarily superior to non-surgical therapy. The purpose of this study was to compare percutaneous orthobiologic treatment to a home exercise therapy program for supraspinatus tears.

**Methods:**

In this randomized-controlled, crossover design, participants with a torn supraspinatus tendon received either ‘BMC treatment’, consisting of a combination of autologous bone marrow concentrate (BMC) and platelet products, or underwent a home exercise therapy program. After three months, patients randomized to exercise therapy could crossover to receive BMC treatment if not satisfied with shoulder progression. Patient-reported outcomes of Numeric Pain Scale (NPS), Disabilities of the Arm, Shoulder, and Hand, (DASH), and a modified Single Assessment Numeric Evaluation (SANE) were collected at 1, 3, 6, 12, and 24 months. Pre- and post-treatment MRI were assessed using the Snyder Classification system.

**Results:**

Fifty-one patients were enrolled and randomized to the BMC treatment group (*n* = 34) or the exercise therapy group (*n* = 17). Significantly greater improvement in median ΔDASH, ΔNPS, and SANE scores were reported by the BMC treatment group compared to the exercise therapy group (-11.7 vs -3.8, *P* = 0.01; -2.0 vs 0.5, *P* = 0.004; and 50.0 vs 0.0, *P* < 0.001; respectively) after three months. Patient-reported outcomes continued to progress through the study’s two-year follow-up period without a serious adverse event. Of patients with both pre- and post-treatment MRIs, a majority (73%) showed evidence of healing post-BMC treatment.

**Conclusions:**

Patients reported significantly greater changes in function, pain, and overall improvement following BMC treatment compared to exercise therapy for high grade partial and full thickness supraspinatus tears.

**Trial registration:**

This protocol was registered with www.clinicaltrials.gov** (**NCT01788683; 11/02/2013).

**Supplementary Information:**

The online version contains supplementary material available at 10.1186/s12891-024-07519-6.

## Introduction

The rotator cuff (RC) is a group of four muscles and tendons, originating at the scapula and inserting onto the superior humeral head, that plays a critical role in stabilizing the glenohumeral joint. RC tears related to trauma or age-related degeneration occur at rates from 4% in patients < 40 years old increasing to more than 50% in those > 60 years old [1, 2]. Conservative management of RC tears typically consists of therapeutic modalities for pain such as ice or heat, oral analgesics or corticosteroids, injected corticosteroids, and/or physical therapy [3] with varying success rates [4–9]. Based on published guidelines, surgical repair is considered necessary for the treatment of high-grade partial thickness and full thickness tears [10], imparting a significant source of societal debility with an annual cost representing billions of dollars in the United States alone [11]. Unfortunately, while the rates of RC surgical repairs have increased [12], evidence indicates that surgery is not necessarily superior to physical therapy [13, 14] especially for partial tears, as have several meta-analyses, which demonstrated patients undergoing surgical treatment often fare no better than those utilizing nonoperative measures alone [15–17].

A potential minimally invasive means of treating RC injury may be found in autologous orthobiologics derived from bone marrow and blood, such as bone marrow concentrate (BMC) platelet-rich plasma (PRP), and platelet lysate (PL). BMC injections have demonstrated encouraging results when utilized in the treatment of some types of RC injury without surgical intervention [18–21]. PRP, meanwhile, has demonstrated early evidence of efficacy in augmenting surgical repair as well as treating painful tendinopathy [22, 23], while PL has shown promise in treating radicular pain [24]. Although exact mechanisms of action are unclear, BMC and PRP likely utilize cellular signaling via cytokines and growth factors to help orchestrate a healing cascade [25–27]. Moreover, BMC contains a population of progenitor cells, referred to as mesenchymal stromal (stem) cells, thought to provide benefits through paracrine mediated effects [28, 29].

The present study represents the culmination of a randomized controlled, crossover trial in which an ultrasound-guided, percutaneous BMC treatment, comprising a combination of autologous BMC and platelet products (PRP and PL), was compared against a home exercise therapy program for the management of non-retracted (high-grade partial-thickness or full-thickness) supraspinatus tears. The primary study objective is the short-term comparison (3-month) of patient reported outcome measures (PROMs) following BMC treatment relative to those undergoing exercise therapy, with those receiving autologous orthobiologics hypothesized to report significantly improved outcomes that are maintained through the 2-year follow-up period.

## Methods

### Patient enrollment

Study participants were recruited from the local community by way of an orthopedic pain practice and through radio, print, website, and social media advertisements from June 2013 to May 2020. Ethics approval for the study protocol was obtained from the International Cellular Medicine Society (OHRP Registration #IRB00002637). Study was registered on ClinicalTrials.gov (NCT01788683) on 11/02/2013. All participants, aged 18–65, provided written informed consent and presented with a non-retracted supraspinatus tendon tear with high T2 signal comprising one-half to full thickness of the tendon thickness in the anterior–posterior and/or superior-inferior planes. Moreover, participants were required to have had continuous pain in the affected shoulder and have previously failed conservative treatment for at least three months. Exclusion criteria are listed in Table 1.

**Table 1:** Exclusion criteria for study enrollment

Exclusion criteria for study enrollment	
• Allergy or intolerance to study medication	• Pregnancy
• Significant bone spur in subacromial space	• Type III acromion
• Glenohumeral osteoarthritis (KL grade II +)	• Chronic opioid use
• Tested positive or been treated for malignancy	• Bleeding disorders • Adhesive capsulitis
• Shoulder instability requiring surgical stabilization	• Grade II + SLAP tear • Contraindications for MRI
• Condition represents a worker’s compensation case	• Massive rotator cuff tear (grade III)
• Quinolone or statin induced myopathy/tendinopathy	• Previous surgery to affected shoulder
• Injection therapies to shoulder within last three months	• Concomitant tears of the bicep tendon
• Currently involved with a health-related litigation procedure	• Symptomatic cervical spine pathology
• Inflammatory or autoimmune based joint disease/pathology • Documented history of drug abuse within six months of treatment	• Severe neurogenic inflammation of the cutaneous nerves about the shoulder
• Currently taking anticoagulants or immunosuppressive medication	

Those enrolled into the study were randomly assigned to undergo either exercise therapy or percutaneous injection of autologous BMC with platelet products (referred to as BMC treatment). The original enrollment protocol randomized between the two groups using a 1:1 ratio, with twenty-five participants in each group. Sample size was determined to have 80% power in detecting a ten-point difference (sd=12.46) in functional outcomes of DASH (α = 0.05, β = 0.2) between the two groups, based on previously published data [30, 31]. However, study enrollment was amended in 2017 to incorporate a 2:1 ratio (34 BMC treatment to 17 exercise therapy) for improved recruitment, after preliminary results showed a larger effect size than anticipated. Sequentially numbered envelopes concealed treatment group allocation based on a computer-generated randomization program and were opened in order of enrollment.

### Initial evaluation

In-office evaluation by a physician was conducted before patient enrollment. Multiplanar MRI images of the injured shoulder were reviewed to assess tear classification. Focusing on the supraspinatus, T2 hyperintensity was used as a hallmark of tendon tears. Further, point-of-care diagnostic ultrasound (Sonosite Edge II Ultrasound System, 13–6 MHz Linear Transducer, FujiFilm, Bothel, WA, USA) was performed, capturing short and long axis views of the supraspinatus tendon in the modified Crass position. Hypoechogenicity within the tendon was used to classify tears as articular-sided, bursal-sided, interstitial, or complete.

### Exercise therapy group

Upon enrollment, patients were notified of the randomized treatment condition. Those assigned to the exercise therapy group met with a physical therapist to receive guidance on a home exercise program, which included an instructional handout and hands-on demonstrations of proper technique. Exercises focused on shoulder strengthening, stability, and stretching. Details of the exercise program have been previously published [18]. Check-in and compliance were monitored at the halfway point (four to six weeks), at which point patients progressed to a second set of exercises for the latter half. At least twelve weeks of self-directed exercise therapy were completed, mirroring studies evaluating the effect of surgical RC repair in comparison to conservative care [8, 32, 33]. Upon completion, if desired improvement had not yet been reached, patients were given the opportunity to cross over to the BMC treatment group.

### BMC treatment group

Patients randomized to the BMC treatment group underwent a blood draw, bone marrow aspiration (BMA), and subsequent percutaneous administration of autologous BMC combined with PRP and PL injectates within a single day. The study utilized aspiration, laboratory cellular processing protocols, and injection techniques previously described [18, 34]. In brief, BMA (60–90 mL) was harvested from six to nine sites along the posterior superior iliac crest, drawing into heparinized syringes (5–15 mL per site). Centrifugation (200xg) yielded a compacted buffy coat (1–3 mL), which was manually isolated as BMC. A small representative volume of BMC (≤ 0.1 mL) was collected from all patients prior to clinical use for cell counting. Red blood cells were removed by osmotic lysis, samples were diluted 100-fold, and an automated cell counter (TC20, BioRad) was used to obtain a nucleated cell count.

An additional volume (~ 60 mL) of peripheral blood was obtained via phlebotomy and processed into PRP using a two-spin technique, first (200xg) to isolate plasma and subsequently (2,300xg) to concentrate platelets in a reduced final plasma volume. Approximately half of the processed PRP was frozen at -80 °C to produce PL. At the time of treatment, ultrasound guidance was utilized to deliver the combination BMC treatment (1–2 mL), containing 60% BMC, 20% PRP, and 20% PL, intratendinously into the visualized supraspinatus tear. If acromioclavicular (AC) or glenohumeral (GH) instability was detected, concentrated PRP will also be injected into the AC or GH ligaments. All patients treated with BMC were given a standard rehab protocol which included having them abstain from activities that caused them to experience pain > 2 out of 10 throughout 12 weeks of rehabilitation. Initially, patients were instructed to perform range of motion exercises 3 × day with applied heat, while avoiding any lifting. Throughout the first month, 3 × daily range of motion and basic strengthening exercises without overhead lifting were recommended. Weeks 5–11, patients were instructed to slowly initiate resistance training. Restrictions were lifted after 12 weeks and patients were guided to introduce eccentric exercises and gradually return to normal activity. Bracing was not utilized. Patients were allowed to receive an additional rotator cuff tendon PRP injection if they reported continued symptoms consistent with RC pathology after BMC treatment.

### Outcome measures

Patient-reported outcome measures (PROMs) including, Numeric Pain Scale (NPS), Disability of the Arm, Shoulder, and Hand (DASH), and a modified Single Assessment Numeric Evaluation (SANE), were collected at baseline and all post-treatment follow-ups (1-, 3-, 6-, 12-, and 24-month). Questions regarding adverse events or complications were also collected at all post-treatment follow-ups. The NPS is an eleven-point scale that asks patients to choose a whole number between zero and ten (0 = no pain; 10 = worst possible pain) that best reflects their pain level over the previous week. The DASH is a thirty-item questionnaire that assesses symptoms, function, and disability of patients with upper extremity musculoskeletal conditions on a scale from zero to one hundred (0 = no disability; 100 = severe disability) [35]. The modified SANE directs patients to indicate on a scale from -100 to + 100 the extent to which they have seen a change in condition compared to before treatment [36]. Change scores (ΔPROMs) were calculated for DASH and NPS by subtracting baseline values from those at the individual follow-up time points. The minimal clinically important difference (MCID) used for the present study was -2 for NPS [37, 38], -10.83 for DASH [39], and 27.25 for SANE [40]. Upon completing the 2-year follow-up, patients could continue providing PROMs on a long-term, annual basis through a patient registry. A final inquiry was conducted to determine how many patients opted for surgical intervention prior to manuscript submittal.

### MRI assessment

MRIs of the injured shoulder were obtained prior to study enrollment and at least 1-year post-BMC treatment to provide an objective measure of healing. A fellowship trained sports medicine physician, blinded to timing of the MRI, interpreted pre- and post-treatment MRIs for all patients via randomized sequence. Fluid-sensitive coronal and sagittal images were graded using the Snyder Classification modified for MRI to characterize the predominant sided tear [41]. This classification system has demonstrated good interrater reliability and MRI correlation to arthroscopy for RC tears [42, 43]. Details are shown in Table 2.

**Table 2:** Modified Snyder Classification system for rotator cuff (RC) tears using MRI

**Snyder Classification System for Rotator Cuff (RC) Tears**
**Location of Tears**
**A** Articular Surface
**B** Bursal Surface
**C** Complete tear connecting A and B side
**Severity of Articular and Bursal RC Tears**
**0** Normal cuff
**I** Minimal, superficial bursal, synovial irritation, slight capsular fraying in a small area, < 1 cm
**II** Fraying/failure of some rotator cuff fibers in along with synovial, bursal, or capsular injury, < 2 cm
**III** More severe rotator cuff injury, including fraying/fragmentation of tendon fibers, often involving the whole surface of a cuff tendon (often the supraspinatus), < 3 cm
**IV** Very severe partial rotator cuff tear that usually contains, including fraying/fragmentation of tendon tissue, sizable flap tear and often encompasses more than a single tendon
**Severity of Complete RC Tears**
**CI** Small, complete tear, such as a puncture wound
**CII** Moderate tear (< 2 cm) that still encompasses only one tendon with no retraction
**CIII** Large, complete tear involving an entire tendon with minimal retraction, usually 3 to 4 cm
**CIV** A massive rotator cuff tear involving two or more tendons, frequently with associated retraction, often L-shaped tear

### Statistical analysis

Patient demographic information and PROMs consisted of both normally and non-normally distributed data sets, and summary statistics were provided as means with standard deviation and medians with interquartile range. Within patient and between patient ΔPROMs three months post-exercise therapy was compared with ΔPROMs three months post-BMC treatment using Wilcoxon matched-pairs signed rank and Mann–Whitney tests, respectively. PROMs were compared over the duration of the study using mixed-effects models with repeated measures design, Geisser-Greenhouse correction, and Tukey multiple comparisons tests. Associations between ΔPROMs and log transformed cell counts were measured using Pearson correlations. Results were considered significant at *P* < 0.05. All statistical analyses were performed using GraphPad Prism 10 (GraphPad Software, Boston, MA).

## Results

**Fig. 1:**
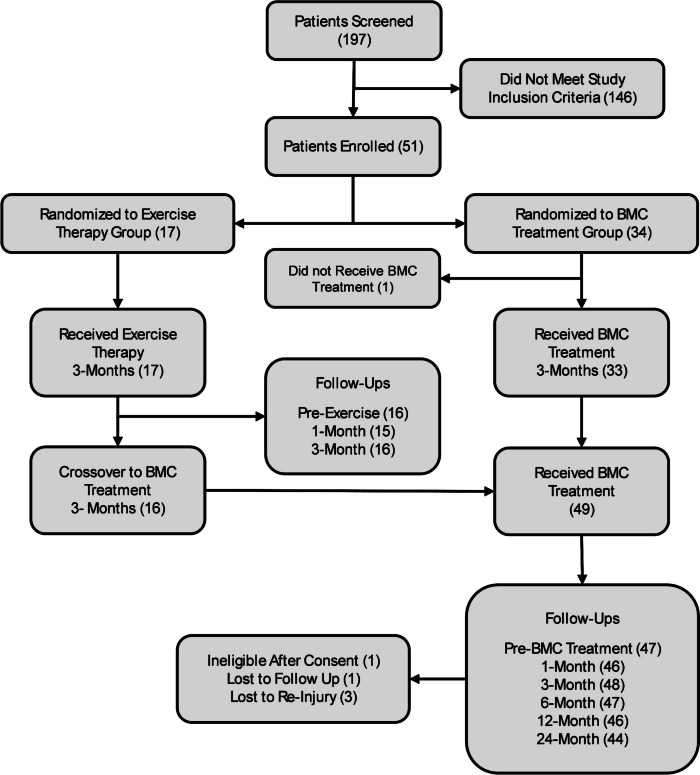
Consort flow diagram

One hundred ninety-seven patients were screened for eligibility, and fifty-one met study criteria and were enrolled, with thirty-four randomized to receive autologous BMC treatment and seventeen randomized to undergo exercise therapy. One patient randomized to receive BMC opted out prior to treatment, one patient in the BMC treatment group was determined to have failed to meet study inclusion requirements post treatment, and one patient initially randomized to exercise therapy did not cross over, therefore was study exited at 6 months (Fig. 1). Patient demographics for all enrolled patients, including age, BMI, height, weight, and nucleated cell counts are shown in Table 3. Significantly more males were randomized into the BMC treatment group compared to females (24 vs 10). Three patients exited the study due to re-injury and one patient converted to surgery (2% surgical rate) during the 2-year follow-up period (Table 4). One patient did not respond to initial BMC treatment and was treated with BMC a second time, approximately 14 months later, and a further nineteen patients received additional PRP injections after (mean = 7.3 months) BMC treatment owing to persistent symptoms consistent with RC pathology.

**Table 3:** Patient demographics provided as mean ± standard deviation (median, interquartile range) separated by treatment group and gender

Demographic	Exercise Therapy		BMC Treatment	
Gender	Female (8)	Male (9)	Female (10)	Male (24)
Age (years)	52 ± 11 (52, 17)	47 ± 12 (46, 20)	54 ± 11 (57, 18)	44 ± 12 (45, 18)
BMI (kg/m^2^)	24.9 ± 5.4 (23.4, 6.5)	28.3 ± 6.1 (26.3, 7.8)	24.8 ± 3.2 (24.5, 4.1)	26.4 ± 3.2 (26.5, 5.0)
Height (inches)	67 ± 3 (67, 7)	71 ± 2 (71, 4)	66 ± 3 (66, 3)	71 ± 3 (70, 3)
Weight (lbs)	157 ± 41 (148, 37)	201 ± 36 (180, 48)	155 ± 30 (153, 33)	188 ± 27 (185, 29)
Cells (millions)	557 ± 249 (593, 513)^a^	864 ± 538 (639, 671)^a^	907 ± 491 (799, 875)	1045 ± 570 (835, 823)

^a^Nucleated cell counts for the exercise therapy group were obtained upon crossover to BMC treatment

**Table 4:** Prevalence of re-injury and RC surgery over the duration of the study period

Follow-Up	Re-Injury	RC Surgery
1-Month	-	-
3-Month	-	-
6-Month	1	-
12-Month	-	-
24-Month	2	1
Total	3	1

One patient required surgical repair following re-injury

Adverse events (AEs) were monitored throughout the study period. There were no serious AEs reported. Five patients who received BMC treatment reported minor AEs, including reports of post-procedural pain at the shoulder and BMA sites and one report of bilateral hand and finger numbness at the 12-month follow-up. Upon clinical review, this subject complained only of discomfort at the distal interphalangeal joints and was assessed as having evidence of finger joint osteoarthritis. Two patients received an injection for capsular distension (at 6 weeks and 4 months). No AEs were reported from the exercise therapy group.

**Fig. 2:**
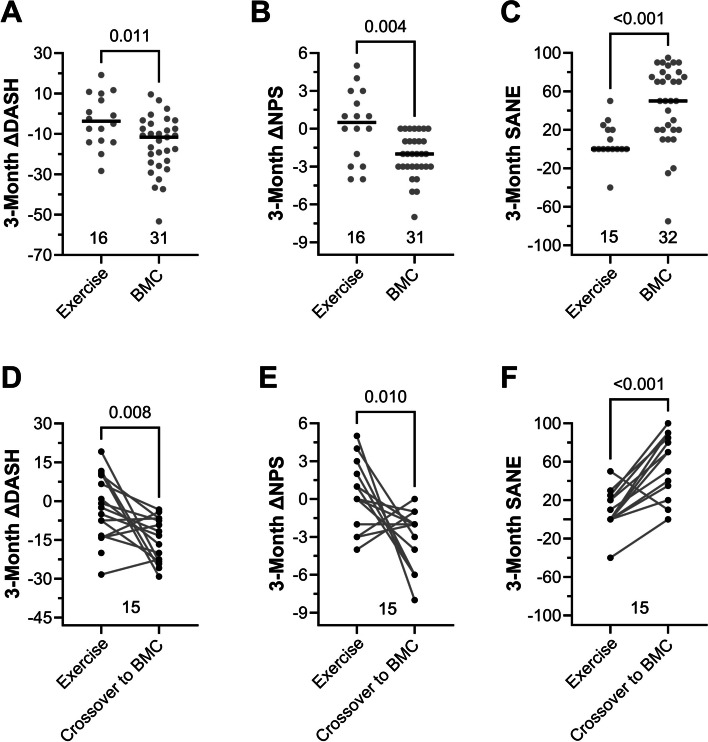
Patient reported outcomes following exercise therapy compared to outcomes following autologous BMC treatment for RC tears. Between group comparisons (**A**-**C**) and within patient (patients in exercise group compared to themselves after crossover to BMC) comparisons (**D**-**F**) of change in function (ΔDASH), pain (ΔNPS), and percent improvement (SANE) scores at the 3-month follow-up timepoint. Horizontal lines represent median values (*n* = displayed)

The primary objective of the present study was to compare change scores from baseline to three months for the BMC treatment group in relation to the exercise therapy group (Fig. 2). The median ΔDASH and ΔNPS scores from the BMC treatment group were significantly lower compared to those from the exercise therapy group at the 3-month follow-up (-11.7 vs -3.8, *P* = 0.01 and -2.0 vs 0.5, *P* = 0.004, respectively) while the median modified SANE score was significantly higher (50.0 vs 0.0, *P* < 0.001) (Fig. 2A-C). By the 3-month follow-up, median ΔDASH, ΔNPS, and SANE scores met or exceeded MCIDs of -10.83, -2, and 27.25, respectively for those in the BMC group, but not for the exercise group. Moreover, within-patient comparisons were performed by comparing ΔPROMs three months after exercise therapy to ΔPROMs three months after crossing over to receive BMC treatment. Significant pairwise decreases in ΔDASH (median difference = -9.2, *P* = 0.008) and ΔNPS (median difference = -3.0, *P* = 0.010), and an increase in SANE (median difference = 50.0, *P* < 0.001) were observed (Fig. 2D-F). One patient originally randomized to the exercise therapy group failed to provide baseline data, preventing calculation of ΔPROMs.

**Fig. 3:**
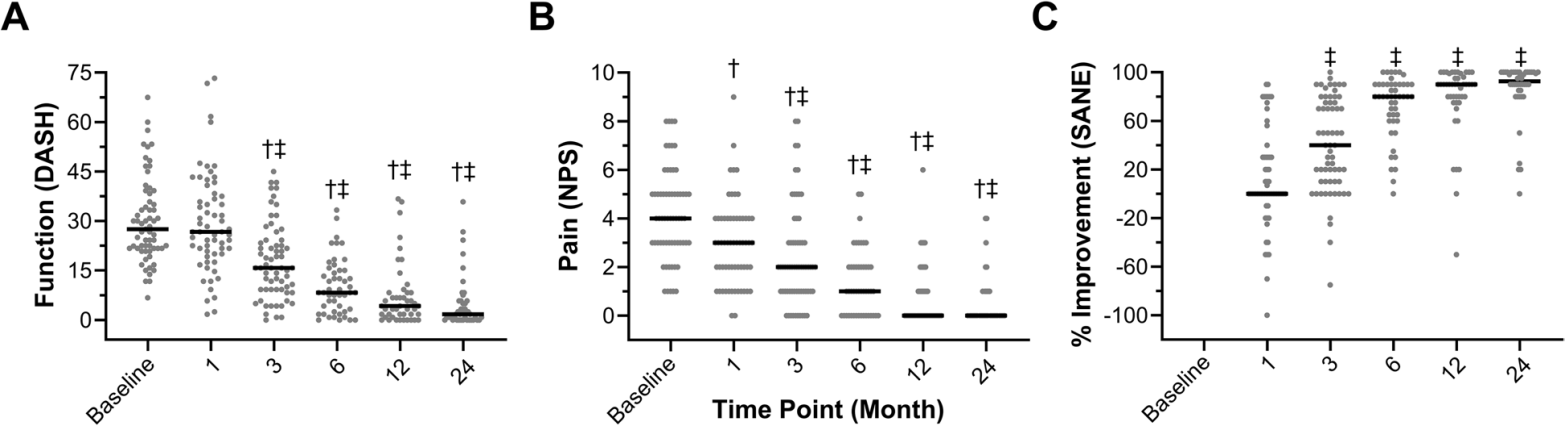
Patient reported outcomes following autologous BMC treatment for RC tears. Shoulder (**A**) function (DASH), (**B**) pain (NPS), and (**C**) percent improvement (SANE) at baseline and 1-, 3-, 6-, 12-, and 24-month follow-up timepoints. Lines represent median values. Significant improvements compared to baseline (†) and 1- month (‡) follow-up timepoints (*p* < 0.05)

PROM scores for BMC treatment and crossover groups continued to improve through the 24-month follow-up (Table 5). There were no significant differences in PROMs between the BMC treatment and the exercise to BMC crossover groups at the 3-month follow-up (DASH *P* = 0.44, NPS *P* = 0.31, SANE *P* = 0.38) nor later at 24-months (DASH *P* = 0.06, NPS = 0.16, SANE = 0.12). Consequently, all patients receiving BMC treatment were included in the analysis of long-term outcomes (Fig. 3). Reported DASH and NPS scores significantly improved from both baseline and 1-month scores (Fig. 3A, B). Similarly, SANE scores significantly improved beyond the 1-month follow-up (Fig. 3C). All PROMS continued to progress significantly beyond the 3- and 6-month follow-ups (Supplementary Tables 1–4). In contrast, DASH and NPS scores did not significantly improve from baseline for the exercise group after three months (DASH *P* = 0.29, NPS *P* = 0.68), and SANE did not significantly improve beyond zero (SANE *P* = 0.20).

**Table 5:** Patient reported outcomes following exercise therapy and/or autologous BMC treatment for RC tears

Follow-Up	DASH	NPS		SANE
**Exercise Therapy**				
Baseline (*n* = 16)	30.8 ± 15.2 (26.3, 27.5)		4.3 ± 1.9 (4.0, 2.8)	-
1-Month (*n* = 15)	26.4 ± 10.9 (24.2, 19.2)		3.5 ± 1.5 (3.0, 2.0)	10.4 ± 24.7 (0, 10)
3-Month (*n* = 16)	27.4 ± 10.7 (23.4, 18.0)		4.5 ± 2.1 (4.5, 3.0)	7.7 ± 20.1 (0, 20)*
**Crossover to BMC Treatment**				
Baseline (*n* = 16)	28.0 ± 10.3 (25.5, 14.0)		4.7 ± 2.1 (4.5, 3.0)	-
1-Month (*n* = 14)	28.2 ± 18.1 (27.5, 18.5)		3.1 ± 1.6 (4.0, 2.3)	17.9 ± 57.9 (10.0, 122.5)
3-Month (*n* = 16)	12.6 ± 8.8 (12.5, 9.2)		1.4 ± 1.4 (1.0, 2.0)	58.8 ± 31.2 (70.0, 47.5)
6-Month (*n* = 16)	9.9 ± 8.4 (7.9, 14.8)		1.3 ± 1.5 (1.0, 2.0)	74.1 ± 22.7 (80.0, 28.7)
12-Month (*n* = 15)	6.7 ± 9.8 (1.7, 8.4)		0.9 ± 1.7 (0.0, 1.0)	82.3 ± 19.8 (90.0, 20.0)
24-Month (*n* = 16)	7.8 ± 10.3 (3.3, 10.0)		0.9 ± 1.5 (0.0, 1.8)	78.7 ± 29.9 (90.0, 19.8)
**BMC Treatment**				
Baseline (*n* = 31)	31.0 ± 13.3 (28.3, 13.3)		3.9 ± 1.7 (4.0, 2.0)	-
1-Month (*n* = 32)	31.2 ± 15.7 (29.6, 20.5)		2.7 ± 1.9 (2.0, 2.0)	10.2 ± 38.7 (0.0, 30.0)
3-Month (*n* = 32)	15.5 ± 10.3 (14.2, 16.3)		1.8 ± 1.5 (2.0, 1.0)	46.6 ± 40.3 (50, 58.8)
6-Month (*n* = 31)	9.7 ± 7.4 (9.2, 11.7)		1.4 ± 1.3 (1.0, 2.0)*	73.2 ± 25.6 (80.0, 25.0)
12-Month (*n* = 31)	7.6 ± 9.4 (4.2, 6.6)		0.7 ± 1.0 (0.0, 1.0)	77.1 ± 34.5 (90.0, 19.0)
24-Month (*n* = 28)	3.4 ± 5.8 (1.3, 4.6)		0.3 ± 0.5 (0.0, 0.0	89.4 ± 19.9 (96.5, 10.0)

Values represent the mean ± standard deviation, (median, interquartile range) and the number of responding patients at each follow-up timepoint. Patient missing a follow-up (*)

**Fig. 4:**
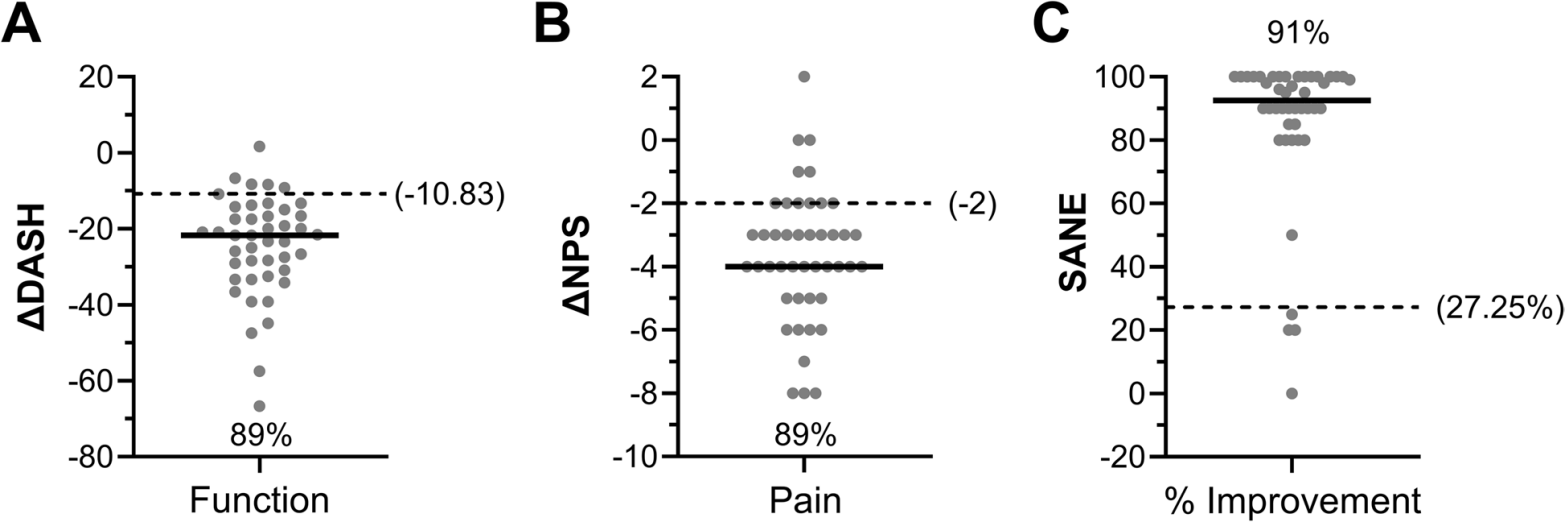
Change in patient reported outcomes following autologous BMC treatment for RC tears in comparison to MCIDs. Solid lines represent the median, and the percentage signifies the portion of patients meeting or exceeding the MCID (dashed lines) for (**A**) function (ΔDASH), (**B**) pain (ΔNPS), and (**C**) percent improvement (SANE) change scores at the 24-month follow-up (*n* = 44)

Twenty-four months following BMC treatment, most patients reported clinically meaningful change in shoulder function, pain, and improvement (Fig. 4). Approximately 90% of responding patients (40 of 44) met or exceeded the DASH MCID of -10.83 (Fig. 4A) and NPS MCID of -2 (Fig. 4B), with more (41 of 44) exceeding the SANE MCID of 27.25 (Fig. 4C). Owing to the disproportionate number of males enrolled in the BMC treatment group, ΔPROMs were compared between genders, and there were no significant differences for ΔDASH, ΔNPS, or SANE (ΔDASH *P* = 0.09, ΔNPS *P* = 0.69, SANE = 0.42). Moreover, patients receiving additional PRP injections (19 of 48) reported lower ΔPROMs, though not significantly, compared to patients having only the initial BMC treatment at the 6-month and 12-month follow-ups, while similar change scores were observed at the final 24-month follow-up (Supplementary Fig. 1).

Long-term PROMs (up to 5 years post-treatment) were available for a subset of patients who continued participating in the patient registry upon study completion. Overall positive results were reported and maintained after the study’s two-year follow-up period (Supplementary Fig. 2). Of the 45 patients receiving BMC treatment and not reporting re-injury within the 2-year study period, 41 (91%) continued to report no surgery prior to publication, while the other 4 could not be reached.

**Fig. 5:**
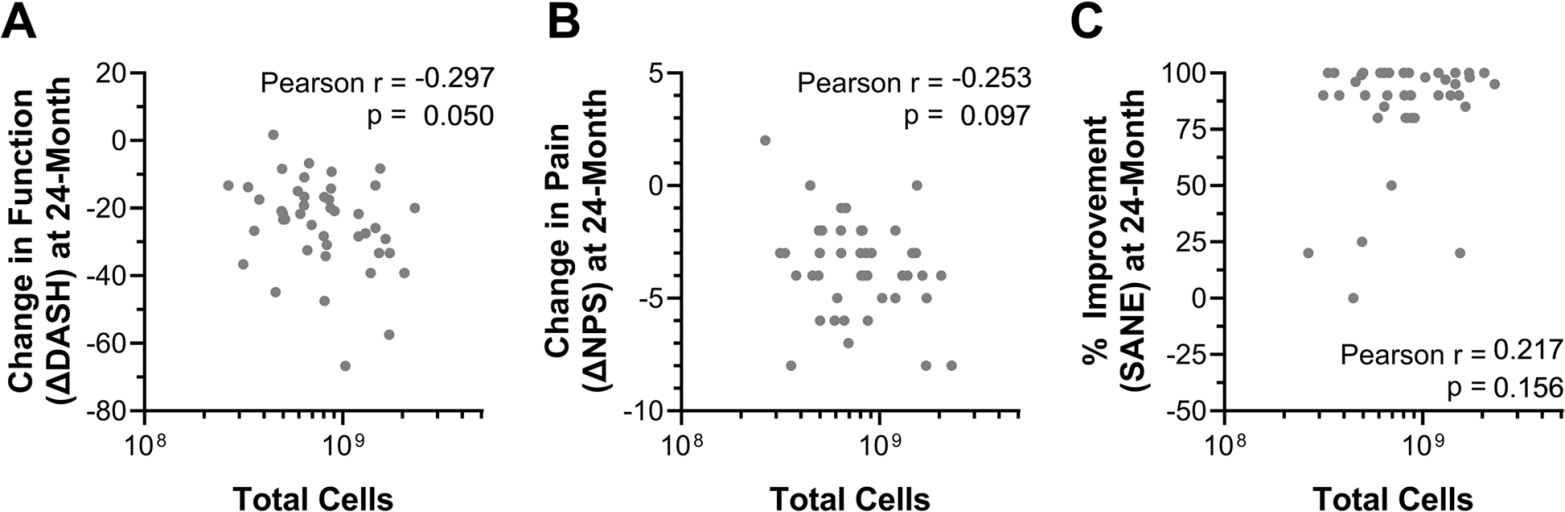
Change in patient reported outcomes following autologous BMC treatment for RC tears versus injectate cellularity. Correlations of 24-month change in (**A**) function (ΔDASH), (**B**) pain (ΔNPS), and (**C**) improvement (SANE) with total nucleated cell counts (*n* = 44)

All patient BMC samples were counted to obtain an estimate of the total nucleated cells in the sample to be injected and to relate the number of cells provided during BMC treatments to long-term ΔPROMs (Fig. 5). A fair correlation was observed between measured nucleated cell counts and ΔDASH scores (*r* = 0.297, *P* = 0.050) at the 24-month follow-up (Fig. 5A), while a weaker trend was observed between cell counts and ΔNPS (*r* = -0.253, *P* = 0.097), albeit not at the established *P* < 0.05 cutoff (Fig. 5B). No relationship was observed between nucleated cell counts and SANE scores (Fig. 5C).

**Fig. 6:**
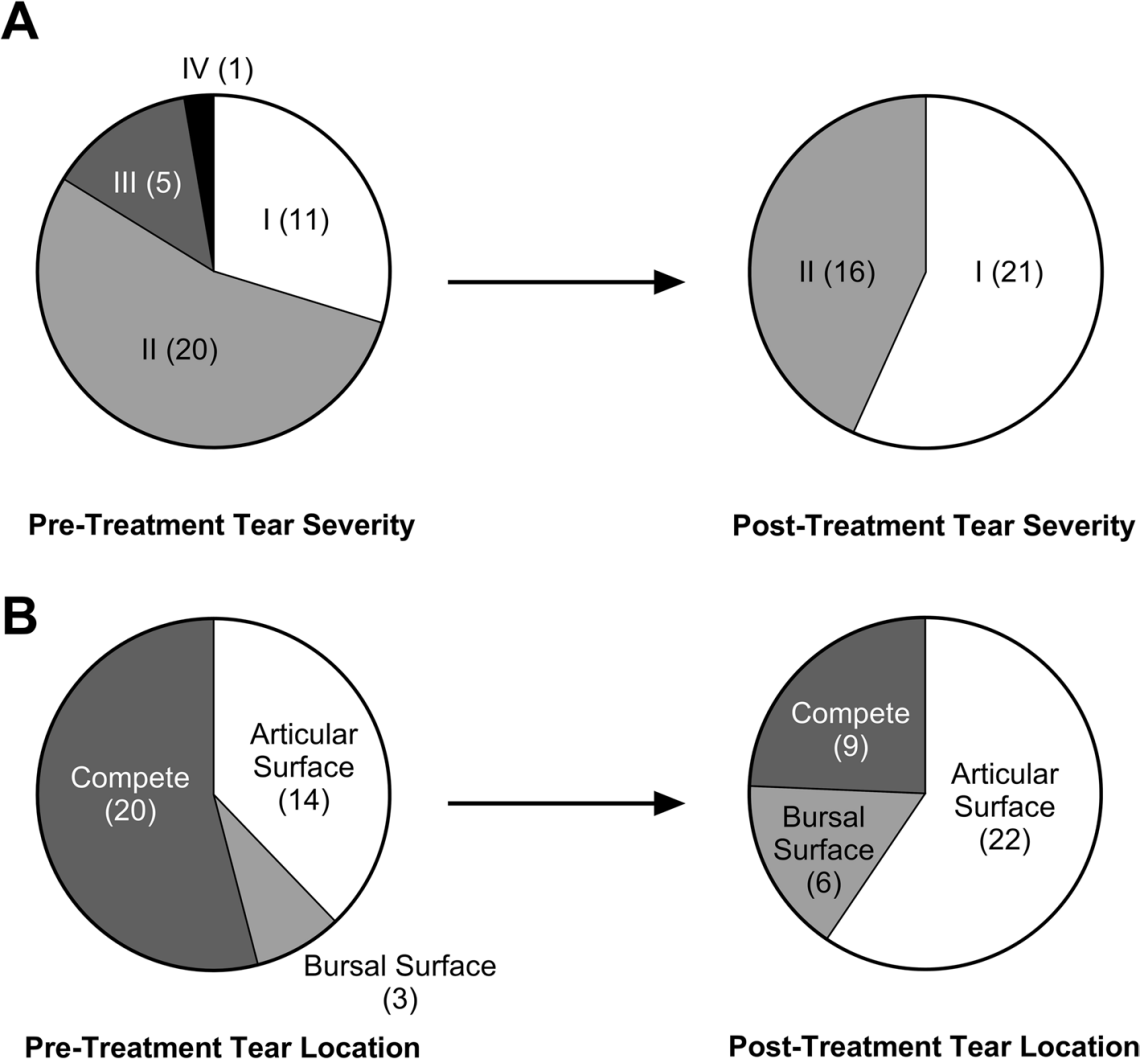
MRI-based modified Snyder Classifications of RC tears. Shoulder MRIs pre- and ≥ 12-months post-BMC treatment (*n* = 37) separated by RC (**A**) tear severity and (**B**) tear location

Pre- and post-procedure shoulder MRIs were available from thirty-seven patients receiving BMC treatment. Using a modified Snyder Classification on the predominant sided tear (articular or bursal) or complete full-thickness tears, baseline RC tear grades ranged from AI through CIII. One year or more following BMC treatment, the majority of patients showed signs of RC tear improvement on MRI, as noted by a decrease in tear severity (e.g. ‘AIII to AI’), a conversion from a complete tear grade to any partial tear grade (e.g. ‘CI to BI’), or a combination thereof (Fig. 6). Overall, RC tear severity and location showed signs of healing on MRI, as grade IV and III tears improved to grade II or I (Fig. 6A) and 11 complete tears improved to partial tears (Fig. 6B). Twenty-seven (73%) patients showed improvement in severity and/or location on the predominate tear, eight patients showed no change and two tears worsened by the post-treatment MRI. Pre- to post-procedure grade changes are shown in Supplementary Fig. 3. Of note, both patients with evidence of worsened post-treatment MRIs demonstrated excellent functional gains and had no reported pain on completion of the study.

## Discussion

The present study represents the culmination of a prospective, randomized controlled, crossover trial comparing percutaneous autologous BMC treatment to exercise therapy for partial or complete non-retracted supraspinatus tendon tears. Patients randomized to the BMC treatment group reported significantly greater improvement in shoulder function (DASH), pain (NPS) and overall improvement (SANE) compared to patients in the exercise therapy group after 3 months, and were sustained through the final follow-up of 24-months after BMC treatment. Patient reported improvements at three months were anticipated based on previous studies assessing tendon healing with orthobiologics [44, 45].

Patients randomized to the exercise therapy group did not initially improve over baseline. The duration for the exercise therapy group was implemented provided previous research showing outcomes three months after exercise to be predictive of longer-term success [46]. Comparisons to physical therapy are common in the rotator cuff surgical literature, lending a useful comparator to BMC treatment in this study design [33]. Additionally, it has been shown that traditional guided physiotherapy is not superior to self-managed exercise in rotator cuff patients [47, 48]. However, this group of patients did not report significant improvements in their condition until after they crossed over and received the BMC treatment.

Cellularity appears to play a contributing role in outcomes afforded by BMC, as a fair correlation was observed between the number of nucleated cells injected and improved functional scores two years after treatment. Recently, the nucleated cell counts of bone marrow aspirates were reported to be positively correlated with both progenitor cell, or MSC, prevalence and concentration, as measured using a colony forming unit (CFU) analysis [49]. Though there are many components of BMC that may contribute to healing, the quantity of progenitor cells has been related to improved musculoskeletal outcomes in previous work by our group and others [50–52]. Another component identified within BMC thought to benefit tissue healing is the anti-inflammatory molecule, interleukin-1 receptor antagonist (IL-1Ra) [53, 54]. In the present study, total nucleated cell counts of the injectate were more strongly associated with improved function than reduced pain. Future research on this topic should look more closely at the cellularity, both in terms of nucleated cells and CFU, and other important biological molecules, such as IL-1RA, as they relate to PROMs following bone marrow-based orthobiologic treatments.

Most patient MRIs (27 of 37) demonstrated evidence of improvement in tear morphology, with some images showing near resolution of their original RC pathology. The natural course expected is progressive enlargement for full-thickness (50% to 82.4%) and partial-thickness tears (13.5% to 26.1%) between 12- and 24-months [44, 55, 56]. Spontaneous improvement or resolution of rotator cuff tears as visualized on MRI is not expected, with low reported rates (4.9% to 11%) [44, 55, 57, 58]. While MRI imaging alone has been shown to poorly correlate with clinically relevant pain [59–61], it is encouraging that imaging did tend to improve following BMC treatment. The Snyder Classification was chosen for directly comparing the predominant sided tear pre- and post-treatment, because it provides a division for both partial and complete tears, something that is lacking with most other rotator cuff grading systems, and it has shown high agreement and accuracy with magnetic resonance arthrography [42].

Several previous studies have demonstrated no significant difference in pain and functional improvements between patients with RC disease who underwent surgery in comparison to conservative care [57, 62]. Despite this, rates of surgical RC repair are steadily increasing [4, 63]. Surgical RC repair is often associated with failure due to re-tear, with reported rates ranging from 11 to 94% [64], as well as complications common to all surgeries such as pneumonia, pulmonary embolism, and infection [63]. By comparison, our data indicates long-term improvement in function and pain after BMC treatment without any unexpected adverse events, supporting the growing literature concluding that autologous orthobiologics injections are likely safe [65–67]. Only a single patient opted for surgery during the course of the study after re-injuring the tendon, and 91% of patients reported no surgeries at > 5 years later. Most, if not all, enrolled patients would have been considered surgical candidates using the current operative strategy of repairing tendons with more than 50% of the tendon thickness [68], similar to our inclusion criteria of requiring at least a 50% tear. An additional MRI review by the senior researcher (CC) noted that 90% of the tears were considered surgical candidates by definition of T2 signal in 100% of the tendon height on coronal image, with or without retraction. Therefore, these data indicate that orthobiologics may be a way to successfully treat common surgical candidates with chronic RC pathology, and a shift in the treatment paradigm could represent a significant amount of savings of cost versus surgical alternatives.

Several study limitations exist. Although the enrolled population included twice as many males as females, this discrepancy may be reflective of the clinical population seeking treatment for shoulder injuries as a previous study documented more than 70% of surgical referrals for RC disorders were males [69]. Outcomes following a self-directed home exercise program may not be representative of a structured, physiotherapist-supervised rehabilitation regimen in a clinical setting. Our study design using exercise therapy as a control group, instead of a sham comparator, was in line with PT control groups in surgical rotator cuff repair studies [32, 33]. Inclusion of an exercise diary or formal physiotherapist-directed exercise could potentially improve compliance for future studies, though we were provided no indication that compliance was not achieved. Additionally, the rehab protocols after BMC treatment were not as rigorous in nature as a guided physiotherapy program nor to the 12-week self-guided exercise program in the control group, but rather acted as a guide to limit initiating strenuous shoulder strength training too fast in their return to activity. As such, it is not believed that these additional instructions impacted the results for the BMC group. This study utilized a combination of BMC, PRP, and PL, and it is not expected that our results would be representative of an injection that included BMC alone. It is possible that PRP and PL had a synergistic effect, increasing the healing response via the supplementation of growth factors, chemokines, and cytokines [70–73]. Likewise, one patient received a second BMC treatment at 14 months due to a lack of initial treatment response. It is not clear what factors may have contributed to the low treatment response, as the nucleated cell count was above average for each BMC treatment. Interestingly, this patient reported 3-year follow-up outcomes of 75% for SANE and 4.2 for DASH, noting significant improvement. Further, some data are missing from our analysis. One patient was lost to follow-up at the 24-month time point, one patient in the exercise therapy group failed to furnish baseline data so ΔPROMs could not be computed, and one patient was determined ineligible, after receiving BMC treatment, due to an underlying cervical pathology.

It is common in orthobiologic treatments for patients to require more than one treatment or injection [74–76]. Given that this protocol only included one BMC injection with PRP, an additional PRP “booster” injection was permitted as this best fit common clinical practice. This also did not require an additional bone marrow aspiration; hence this was less invasive than the original procedure. The addition of PRP booster injections brought ΔPROMs in line with those patients not receiving boosters by the 24-month follow-up.

This study suggests that non-surgical treatments such as BMC with platelet injection are a viable alternative treatment option for rotator cuff tears. Further randomized trials using BMC injections with larger samples of patients and blinded comparative control groups are needed. Other advances in technology for rotator cuff rehabilitation should also be considered [77].

## Conclusion

To our knowledge, this is the first randomized controlled trial of partial and complete supraspinatus tears assessing patient response and imaging changes to the tendon in the nonsurgical setting. This study demonstrates that BMC with platelets injection significantly improved DASH, NPS and SANE scores versus self-guided exercise therapy at 3-months for rotator cuff patients, with sustained effects through 2-year follow-up.

### Supplementary Information

Supplementary Material 1.

Supplementary Material 2.

Supplementary Material 3.

Supplementary Material 4.

Supplementary Material 5.

Supplementary Material 6.

Supplementary Material 7.

## Data Availability

Data used in this analysis during the current study may be made available upon reasonable request from the corresponding author.
