# Supplementary material for: Percutaneous bone marrow concentrate and platelet products versus exercise therapy for the treatment of rotator cuff tears: a randomized controlled, crossover trial with 2-year follow-up
Source: BMC Musculoskelet Disord. 2024 May 18;25:392. doi: 10.1186/s12891-024-07519-6 (PMC11102209; doi:10.1186/s12891-024-07519-6)
Supplement: Supplementary file 1 — Supplementary Material 1. [file 12891_2024_7519_MOESM1_ESM.docx]

**Supplementary Table 1-** Patient reported outcomes scores following autologous BMC treatment for RC tears.

| **Follow-Up** | **DASH** | **NPS** | **SANE** |
| --- | --- | --- | --- |
| Baseline (n=47) | 30.0 ± 12.3 (27.5, 13.3) | 4.2 ± 1.9 (4.0, 2.0) | - |
| 1-Month (n=46) | 30.3 ± 16.3 (28.8, 17.9) | 2.8 ± 1.8 (2.5, 3.0) | 12.5 ± 44.9 (0.0, 45.0) |
| 3-Month (n=48) | 14.5 ± 9.9 (13.8, 13.4) | 1.7 ± 1.5 (1.5, 1.0) | 50.7 ± 37.6 (60.0, 60.0)* |
| 6-Month (n=47) | 9.8 ± 7.7 (8.3, 12.5) | 1.4 ± 1.4 (1.0, 2.0)* | 73.5 ± 24.4 (80.0, 25.0) |
| 12-Month (n=46) | 7.3 ± 9.5 (4.2, 6.8) | 0.8 ± 1.3 (0.0, 1.0) | 78.8 ± 30.3 (90.0, 18.0) |
| 24-Month (n=44) | 5.0 ± 7.9 (1.7, 5.8) | 0.5 ± 1.0 (0.0, 1.0) | 85.5 ± 24.2 (92.5, 15.0) |

Values are the mean ± standard deviation (median, interquartile range) and the number of responding patients at each follow-up timepoint. Patient missing a follow-up (*).
