# Supplementary material for: Percutaneous bone marrow concentrate and platelet products versus exercise therapy for the treatment of rotator cuff tears: a randomized controlled, crossover trial with 2-year follow-up
Source: BMC Musculoskelet Disord. 2024 May 18;25:392. doi: 10.1186/s12891-024-07519-6 (PMC11102209; doi:10.1186/s12891-024-07519-6)
Supplement: Supplementary file 2 — Supplementary Material 2. [file 12891_2024_7519_MOESM2_ESM.docx]

**Supplementary Table 2** – Mean differences, 95% confidence intervals, and adjusted P values for DASH score comparisons over 2-year follow-up.

| **Follow-Up** | **Mean Difference** | **95% CI** | **Adjusted P Value** |
| --- | --- | --- | --- |
| Baseline vs. 1 Month | 0.3 | -6.2 to 6.7 | >0.999 |
| Baseline vs. 3 Month | -15.4 | -20.7 to -10.1 | <0.001 |
| Baseline vs. 6 Month | -20.2 | -25.3 to -15.1 | <0.001 |
| Baseline vs. 12 Month | -22.6 | -28.7 to -16.6 | <0.001 |
| Baseline vs. 24 Month | -25.0 | -30.9 to -19.1 | <0.001 |
| 1 Month vs. 3 Month | -15.7 | -21.7 to -9.7 | <0.001 |
| 1 Month vs. 6 Month | -20.5 | -27.1 to -13.9 | <0.001 |
| 1 Month vs. 12 Month | -22.9 | -30.7 to -15.1 | <0.001 |
| 1 Month vs. 24 Month | -25.3 | -33.5 to -17.1 | <0.001 |
| 3 Month vs. 6 Month | -4.8 | -8.0 to -1.6 | <0.001 |
| 3 Month vs. 12 Month | -7.2 | -12.0 to -2.4 | <0.001 |
| 3 Month vs. 24 Month | -9.6 | -14.4 to -4.8 | <0.001 |
| 6 Month vs. 12 Month | -2.4 | -5.4 to 0.6 | 0.176 |
| 6 Month vs. 24 Month | -4.8 | -8.0 to -1.6 | <0.001 |
| 12 Month vs. 24 Month | -2.4 | -5.2 to 0.4 | 0.132 |
