# Supplementary material for: Percutaneous bone marrow concentrate and platelet products versus exercise therapy for the treatment of rotator cuff tears: a randomized controlled, crossover trial with 2-year follow-up
Source: BMC Musculoskelet Disord. 2024 May 18;25:392. doi: 10.1186/s12891-024-07519-6 (PMC11102209; doi:10.1186/s12891-024-07519-6)
Supplement: Supplementary file 3 — Supplementary Material 3. [file 12891_2024_7519_MOESM3_ESM.docx]

**Supplementary Table 3** – Mean differences, 95% confidence intervals, and adjusted P values for NPS score comparisons over time.

| **Follow-Up** | **Mean Difference** | **95% CI** | **Adjusted P Value** |
| --- | --- | --- | --- |
| Baseline vs. 1 Month | -1.3 | -2.2 to -0.5 | <0.001 |
| Baseline vs. 3 Month | -2.5 | -3.4 to -1.6 | <0.001 |
| Baseline vs. 6 Month | -2.8 | -3.6 to -2.0 | <0.001 |
| Baseline vs. 12 Month | -3.4 | -4.2 to -2.5 | <0.001 |
| Baseline vs. 24 Month | -3.6 | -4.6 to -2.7 | <0.001 |
| 1 Month vs. 3 Month | -1.1 | -1.9 to -0.4 | <0.001 |
| 1 Month vs. 6 Month | -1.5 | -2.2 to -0.7 | <0.001 |
| 1 Month vs. 12 Month | -2.0 | -3.0 to -1.1 | <0.001 |
| 1 Month vs. 24 Month | -2.3 | -3.2 to -1.4 | <0.001 |
| 3 Month vs. 6 Month | -0.3 | -0.9 to 0.2 | 0.544 |
| 3 Month vs. 12 Month | -0.9 | -1.6 to -0.2 | 0.006 |
| 3 Month vs. 24 Month | -1.2 | -2.0 to -0.4 | 0.001 |
| 6 Month vs. 12 Month | -0.6 | -1.0 to -0.1 | 0.011 |
| 6 Month vs. 24 Month | -0.8 | -1.5 to -0.2 | 0.005 |
| 12 Month vs. 24 Month | -0.3 | -0.8 to 0.2 | 0.571 |
