# Supplementary material for: Percutaneous bone marrow concentrate and platelet products versus exercise therapy for the treatment of rotator cuff tears: a randomized controlled, crossover trial with 2-year follow-up
Source: BMC Musculoskelet Disord. 2024 May 18;25:392. doi: 10.1186/s12891-024-07519-6 (PMC11102209; doi:10.1186/s12891-024-07519-6)
Supplement: Supplementary file 4 — Supplementary Material 4. [file 12891_2024_7519_MOESM4_ESM.docx]

**Supplementary Table 4** – Mean differences, 95% confidence intervals, and adjusted P values for SANE score comparisons over time.

| **Follow-Up** | **Mean Difference** | **95% CI** | **Adjusted P Value** |
| --- | --- | --- | --- |
| 1 Month vs. 3 Month | 38.1 | 23.7 to 52.6 | <0.001 |
| 1 Month vs. 6 Month | 60.9 | 43.4 to 78.5 | <0.001 |
| 1 Month vs. 12 Month | 66.3 | 46.2 to 86.4 | <0.001 |
| 1 Month vs. 24 Month | 73.0 | 50.6 to 95.3 | <0.001 |
| 3 Month vs. 6 Month | 22.8 | 9.7 to 35.9 | <0.001 |
| 3 Month vs. 12 Month | 28.2 | 12.1 to 44.2 | <0.001 |
| 3 Month vs. 24 Month | 34.9 | 16.4 to 53.3 | <0.001 |
| 6 Month vs. 12 Month | 5.4 | -3.9 to 14.6 | 0.478 |
| 6 Month vs. 24 Month | 12.1 | -0.01 to 24.1 | 0.050 |
| 12 Month vs. 24 Month | 6.7 | -5.73 to 19.1 | 0.545 |
