# Supplementary material for: Percutaneous bone marrow concentrate and platelet products versus exercise therapy for the treatment of rotator cuff tears: a randomized controlled, crossover trial with 2-year follow-up
Source: BMC Musculoskelet Disord. 2024 May 18;25:392. doi: 10.1186/s12891-024-07519-6 (PMC11102209; doi:10.1186/s12891-024-07519-6)
Supplement: Supplementary file 5 — Supplementary Material 5. [file 12891_2024_7519_MOESM5_ESM.pptx]

## Slide 1
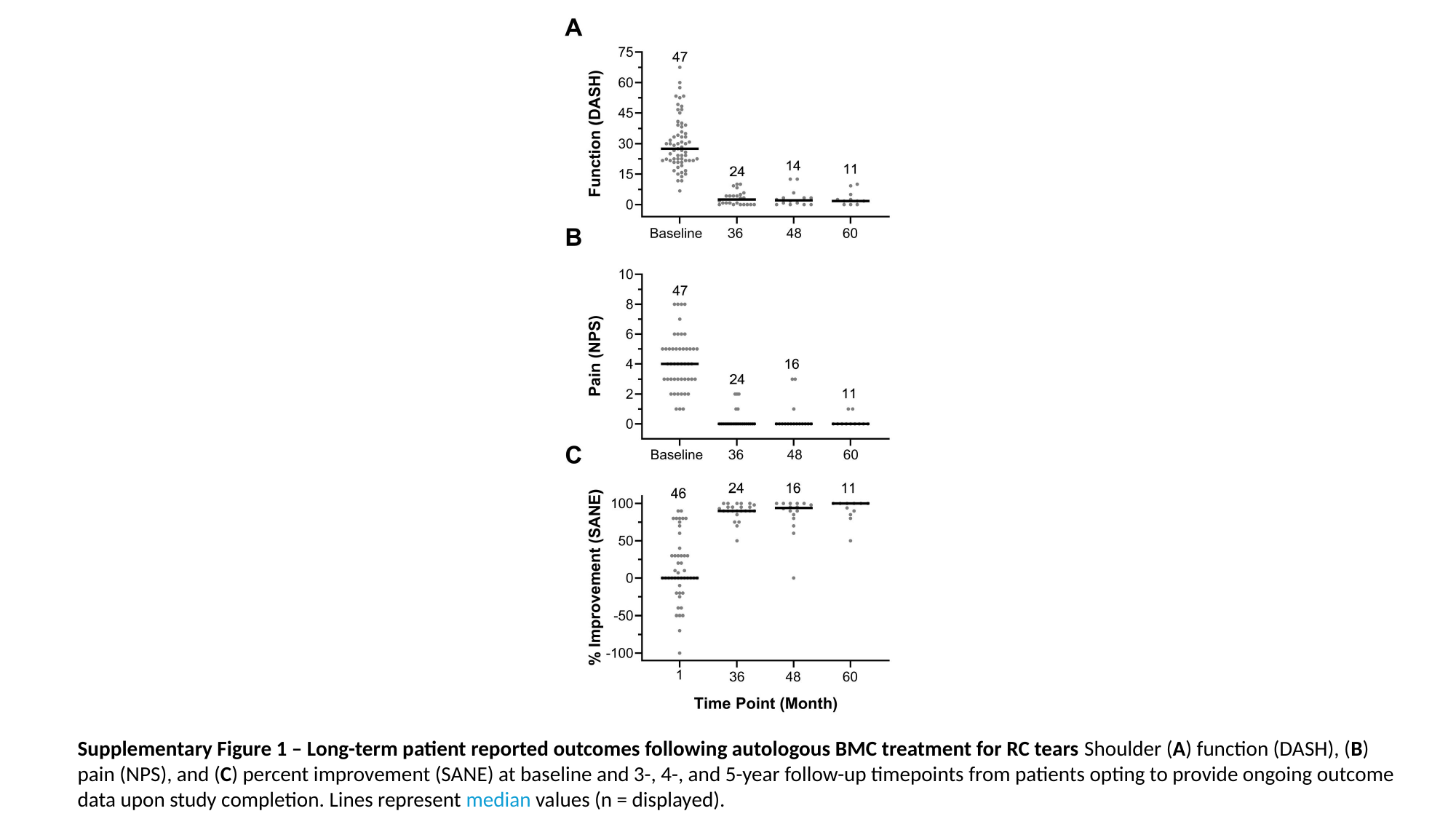

Supplementary Figure 1 – Long-term patient reported outcomes following autologous BMC treatment for RC tears Shoulder (A) function (DASH), (B) pain (NPS), and (C) percent improvement (SANE) at baseline and 3-, 4-, and 5-year follow-up timepoints from patients opting to provide ongoing outcome data upon study completion. Lines represent median values (n = displayed).
