# Supplementary material for: Percutaneous bone marrow concentrate and platelet products versus exercise therapy for the treatment of rotator cuff tears: a randomized controlled, crossover trial with 2-year follow-up
Source: BMC Musculoskelet Disord. 2024 May 18;25:392. doi: 10.1186/s12891-024-07519-6 (PMC11102209; doi:10.1186/s12891-024-07519-6)
Supplement: Supplementary file 6 — Supplementary Material 6. [file 12891_2024_7519_MOESM6_ESM.pptx]

## Slide 1
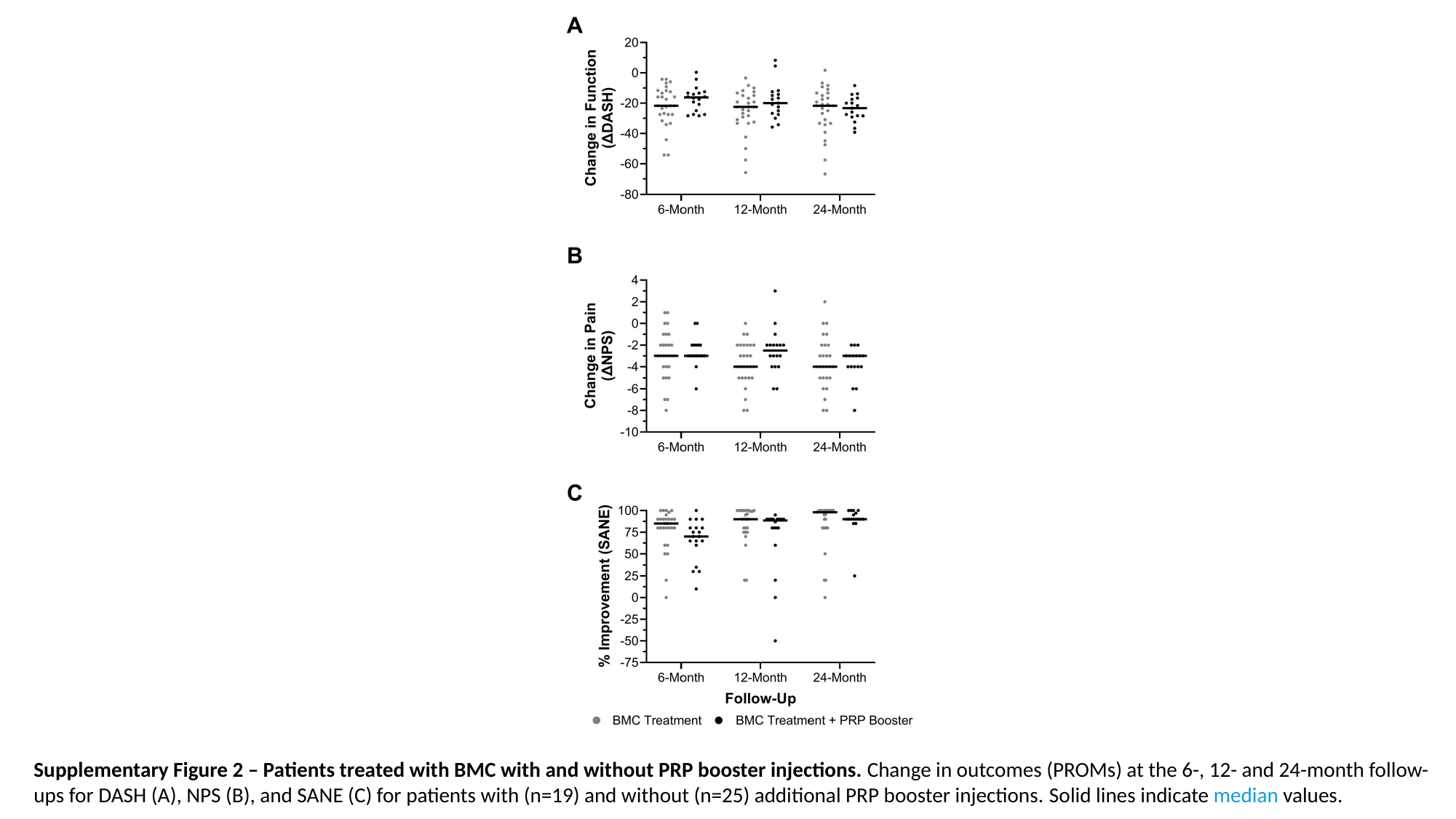

Supplementary Figure 2 – Patients treated with BMC with and without PRP booster injections. Change in outcomes (PROMs) at the 6-, 12- and 24-month follow-ups for DASH (A), NPS (B), and SANE (C) for patients with (n=19) and without (n=25) additional PRP booster injections. Solid lines indicate median values.
