# Supplementary material for: Percutaneous bone marrow concentrate and platelet products versus exercise therapy for the treatment of rotator cuff tears: a randomized controlled, crossover trial with 2-year follow-up
Source: BMC Musculoskelet Disord. 2024 May 18;25:392. doi: 10.1186/s12891-024-07519-6 (PMC11102209; doi:10.1186/s12891-024-07519-6)
Supplement: Supplementary file 7 — Supplementary Material 7. [file 12891_2024_7519_MOESM7_ESM.pptx]

## Slide 1
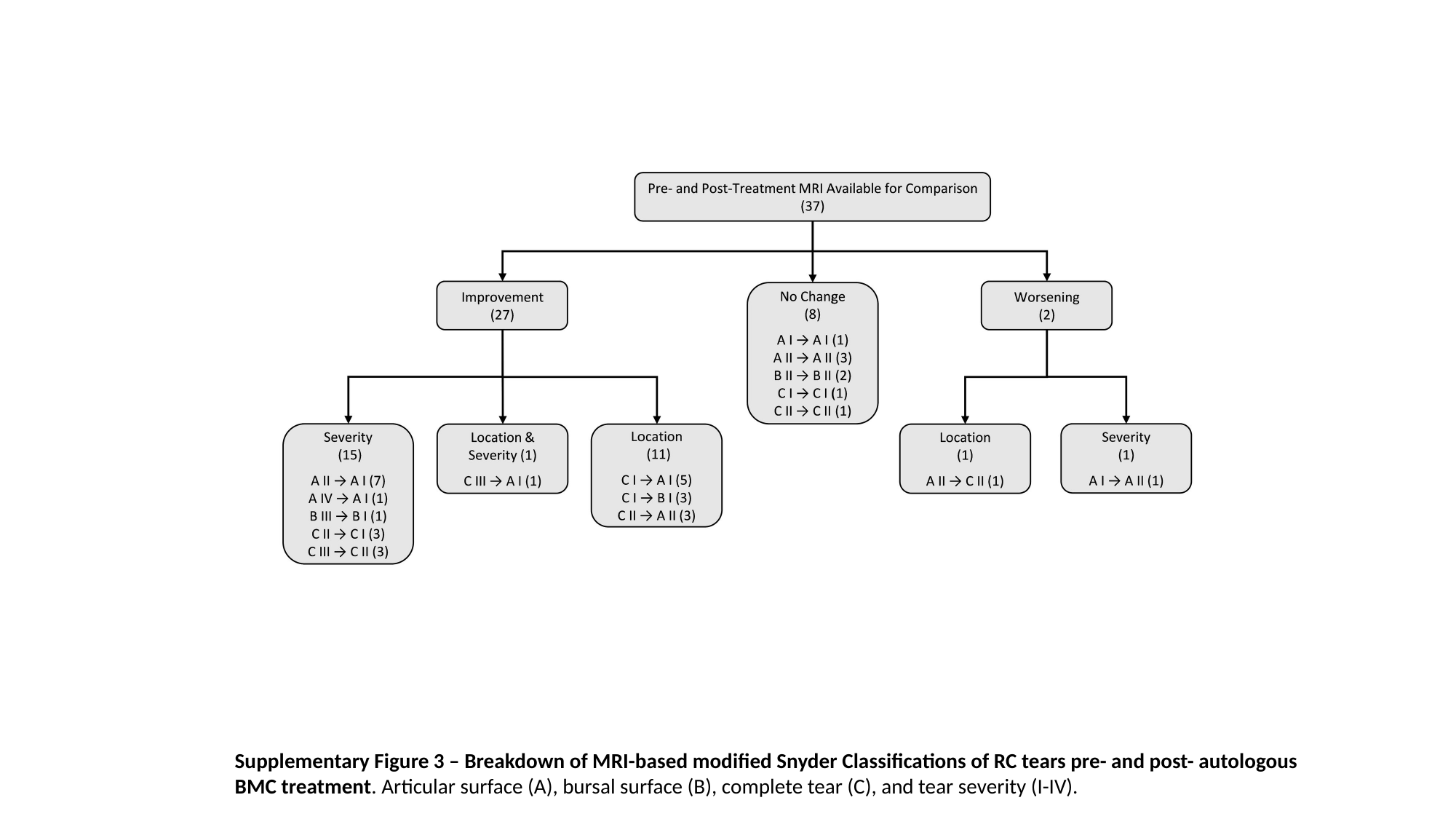

Supplementary Figure 3 – Breakdown of MRI-based modified Snyder Classifications of RC tears pre- and post- autologous BMC treatment. Articular surface (A), bursal surface (B), complete tear (C), and tear severity (I-IV).
